# Developing cartoons for long-term condition self-management information

**DOI:** 10.1186/1472-6963-14-60

**Published:** 2014-02-08

**Authors:** Anne Kennedy, Anne Rogers, Christian Blickem, Gavin Daker-White, Robert Bowen

**Affiliations:** 1 Faculty of Health Sciences, NIHR CLAHRC Wessex, University of Southampton, Southampton, UK; 2 Centre for Primary Care, Institute of Population Health, University of Manchester, Manchester, UK; 3 NIHR Greater Manchester Primary Care Patient Safety Translational Research Centre, University of Manchester, Manchester, UK

**Keywords:** Patient information, Cartoons, Health literacy, Self-management, Long-term conditions

## Abstract

**Background:**

Advocating the need to adopt more self-management policies has brought with it an increasing demand for information about living with and making decisions about long-term conditions, with a significant potential for using cartoons. However, the purposeful use of cartoons is notably absent in many areas of health care as is evidence of their acceptability to patients and lay others. This paper outlines the process used to develop and evaluate cartoons and their acceptability for a series of self-management guidebooks for people with inflammatory bowel disease, irritable bowel syndrome, diabetes, chronic obstructive pulmonary disease and chronic kidney disease (CKD).

**Methods:**

Principles for a process to develop information and cartoons were developed. Cartoon topics were created using qualitative research methods to obtain lay views and experiences. The CKD guidebook was used to provide a detailed exemplar of the process. Focus group and trial participants were recruited from primary care CKD registers. The book was part of a trial intervention; selected participants evaluated the cartoons during in-depth interviews which incorporated think-aloud methods.

**Results:**

In general, the cartoons developed by this process depict patient experiences, common situations, daily management dilemmas, making decisions and choices and the uncertainties associated with conditions. CKD cartoons were developed following two focus groups around the themes of getting a diagnosis; understanding the problem; feeling that facts were being withheld; and setting priorities. Think-aloud interviews with 27 trial participants found the CKD cartoons invoked amusement, recognition and reflection but were sometimes difficult to interpret.

**Conclusion:**

Humour is frequently utilised by people with long-term conditions to help adjustment and coping. Cartoons can help provide clarity and understanding and could address concerns related to health literacy. Using cartoons to engage and motivate people is a consideration untapped by conventional theories with the potential to improve information to support self-management.

## Background

Maximising the acceptability and utility of information for use by patients has become a central feature of attempts to improve the quality and engagement of patients in the field of self-management [1]. Cartoons have been used in therapeutic encounters to promote the understanding and articulation of emotional difficulties and promote engagement with talking therapies (where people explore feelings and thoughts with a therapist) [2] and thus potentially might contribute to the production and utilisation of information for self-management support. There is evidence of cartoons being viewed as an effective means of communicating important medical and health concerns. For example in the US a political and social critique formed the bases of cartoons which provided the public with a focus on the opportunities for preventing illness and accidents [3]. However, more generally the use of cartoons remains the exception rather than a rule and a marginalised activity viewed as quirky rather than a mainstream form of information communication, packaging and delivery [4]. In long-term condition management, where the demand for information by patients and the public is increasing but evidence of the effectiveness of written information on its own to radically change behaviour is equivocal [4], we argue that the use of cartoons has significant potential.

It is worth noting that to date the primary focus of the dissemination of information has been orientated to making language clearer and more user friendly [5]. Increasing treatment burden for patients with complex health and social problems suggests the need to strategically develop material in a way which is ‘minimally disruptive’ [6]. Cartoons offer a potentially more normalised and accessible way of engaging with self-management options than some established therapies because of their humorous associations with everyday dilemmas. Cartoons are part of everyday life so they provide a normalised connecting point of visual communication and continuity during the biographical reconstruction necessary to adapt to living with a long-term condition [7]. However, this leaves open the question of what might need to be done in addition, i.e. using different media. How patients relate to others through informational media is important particularly through identification with helpful or unhelpful thoughts and beliefs (this requires encapsulating patient experience differently). This has been found to be important in both communication and patient activation relating to self-management activities. It may also be the case that cartoons can act as a resource for others in need of practical help to explain the lay actualization of everyday self-care resources (such as how to engage with other people, food information and local clubs and activities). There is a strong driver to reciprocate in chronic illness and to be seen to be of use to others [8,9]; to which users can help through participating in a process of developing user focused information.

This paper is a description of the process of involving patients in developing cartoons for self-management guidebooks and a reflection on the use of humour and metaphor intrinsic to accompanying information conveyed by cartoons.

### Humour for communication and promoting self-management

Humour is used a great deal in everyday communication and surveys of public health and patient information show that cartoons are used to amuse or inform through several formats (written, audio-visual or online media). However, it is less clear how actual cartoons are developed or how they can be used to the greatest effect. In particular, there is little literature concerning the rationale behind the use of cartoons or visual images in patient information. However, there is a recognition of the need to develop an evidence base so that images in health care can be used to ‘maximise good and minimize harm’ [10] and that the potential to alienate or disempower is recognised [7]. Conventional metaphors are made real in cartoons (e.g. expression of emotions and feelings). Illness has also been powerfully conveyed as metaphor [11] suggesting a vehicle in the translation, conveyance and sharing of health information via cartoons and humour which perhaps provide an even more powerful hook [12].

People with long-term conditions and their significant others often use humour as a way of coping with the associated stresses, anxieties and embarrassments of their lives [13-19]. This is rarely acknowledged as a formal self-management strategy but seems linked to the ontological desire to continue with living life despite having to put up with the vagaries of dealing continuously with a long-term condition [6]. Humour works for people on a number of levels. It can be a source of resilience. Recent empirical research has found that humour serves a number of functions in social relationships including affiliation, deflecting attention from the self, as a protection in risky circumstances, reduction of discomfort, avoiding embarrassment, maintaining a light-hearted outlook, amusement and breaking up monotony [20]. It is relevant then to acknowledge and recognise these functions of humour in developing information resources. Whilst studies which have explored the role of humour have aided understanding of reactions to health events, the therapeutic value and utility for self-management might be captured and used through the medium of cartoons. For example, for people with irritable bowel syndrome (a condition which is experienced as embarrassing) humour appears to be valued as a preferred and officially unrecognised strategy for management which does not appear in formal self-management strategies such as action planning [19]. Men with cancer have been found to use humour and jokes to manage feelings and reduce tensions [13].

In a review of the purpose and function of humour in health [14], three theories of how humour and health might be linked are explored: Superiority Theory or Tendentious or Disparagement Theory (Hobbes 1588–1679): An aggressive form of humour which takes pleasure in others’ failings or discomfort, this includes self-deprecating humour used against the self; Incongruity Theory (Kant 1724–1804): Humour where the punch line or resolution is inconsistent or incongruous with the set-up; and Relief or Release Theory (Freud 1856–1938): Humour released by ‘excess’ nervous energy which actually masks other motives or desires.

Humour and laughter can be seen as having a direct and physiological effect on health, as well as an indirect effect by enhancing coping abilities so moderating stress. McCreaddie and Wiggins [14] found limited evidence on the direct effects of humour but wider acceptance of the evidence for indirect effects. They question whether certain types of humour, in particular the self-depreciation widely used by patients, is adaptive (indicative of good social skills and ability to cope) or maladaptive (indicative of self-loathing). A further utility of humour in the management of long-term conditions relates to a link between humour, hierarchy and subversion. In the context of interprofessional relationships humour has been associated with a strategy used by rank and file members to resist and attenuate instructions coming from powerful professionals [21]. In the context of self-management, which can be construed in a part as delegated work to patients, there is a similar potential means by which humour can be used by patients to subvert, challenge or resist the instructions of health professionals which at the same time enables an empowering and positive means of being able to self-manage. A less subservient approach to professionals and strategic non-compliance have been identified as essential elements in achieving a balance in a person’s life and attaining a sense of well-being in managing diabetes [22].

One purpose of cartoons is to help provide clarity, insight and understanding [23]. The use of pictures or cartoons in patient information has been shown to be more effective than using text alone [15,24-26]. This has most salience where health literacy is conceptualised as a personal asset, orientated to developing skills and capacities which enable individuals to exert more mastery and control over their health and the factors that influence health and illness [27]. Non-text-based approaches or visual methods including collage, photo-elicitation (where photos are used as part of an interview), self-portraits and other drawing-based activities such as relational maps and timelines are increasingly used in research. Drawings have been used as an insightful research method to explore the ways in which people understand and make sense of their illness conditions, in particular for people with low levels of literacy [28]. Here we extended this rationale to the development of information resources for long-term conditions.

One of the specific purposes for developing cartoons to accompany self-management information could be to address concerns related to health literacy (conceptualised as a risk and asset) [27]. We see the use of cartoons as addressing health literacy in a number of ways: To impart humour (which improves recall and understanding of information) [24]; to provide resonance with shared experiences (a visual reminder that other people have similar problems and have found solutions); to communicate complex ideas (which often take a lot of words to explain and a cartoon may provide a simplified short-cut); and to illustrate, illuminate and show ways of dealing with embarrassing situations (people unable to read about other people’s health problems may feel so embarrassed about their symptoms that they are unable to talk about them – a cartoon showing such symptoms might be enough to prompt help-seeking). Cartoons can be used to present situations in a non-threatening way and their simplicity can give a clear focus to an idea or thought; it has been found that photos can be confusing when there is too much detail [24].

It is recognised that patients and recipients of information should be consulted to ensure that cartoons or pictures are meaningful and not used in a way which is counter-productive [24]. However, there is currently nothing in the literature which explores how ideas for cartoons can be generated and translated in a collective and reciprocal way to engender engagement with self-management support. It appears that in the main, the ideas result from clinicians and researchers who commission illustrators, but it is unclear whether or how patients’ voices or experiences are incorporated into the ideas for translating messages and ideas for support for living with a long-term condition.

The research question of interest to us is: Can patient experiences and views be translated into cartoons which are acceptable and useful in self-management information? Here we start by outlining an approach to use lay views and patient experiences to create cartoons during the development of self-management guidebooks. We then use the most recently developed guidebook for people with early stage chronic kidney disease (CDK) as an exemplar of the methods used to develop and refine cartoons and as the subject for a qualitative evaluation of their impact.

### Outline of an approach using patient experiences to develop cartoons

Over the past two decades, we have refined an approach to develop cartoons for use in patient information. A series of self-management guidebooks have been written with and for people with inflammatory bowel disease, irritable bowel syndrome, diabetes, chronic obstructive pulmonary disease and CKD [29-33]. In the guidebooks, lay informed experiences have been given equal weight to medically and clinically informed evidence. To collect and synthesise views and experiences we used qualitative methods (focus groups) and thematic analysis to generate topics and themes. We tapped into the core feature of the focus group process: the interaction between participants was used to generate a collective voice that could translate into the development of a cartoon image [34]. The cartoons were intended to encourage engagement with self-management and convey the universality of daily dilemmas and uncertainties of living with a long-term condition to patients to highlight the relevancy of the information.

The cartoon development drew on discussions about patient practices and experiences concerning: the experience of living with the condition, common situations, dilemmas of day-to-day management, the opportunities and difficulties of making decisions or choices, or the uncertainties associated with the condition. For each booklet, a list of topics for cartoons was developed by the research team using the empirical data and linked to a patient quote if possible. A cartoonist willing to take on the brief was found and an iterative process was used to refine the cartoons.

### An exemplar of the approach: the ‘keeping your kidneys healthy’ guidebook

This exemplar is used because it is the most recent guidebook using the most refined methodology. The development and trial were part of the NIHR CLAHRC (Collaboration for Leadership in Applied Health Research and Care) for Greater Manchester; a five year programme of research which commenced in 2008 aiming to improve health care and reduce inequalities in health for people living with chronic vascular conditions.

Ethical research approval was obtained (NRES Committee NorthWest Greater Manchester Central REC reference:11/NW0855).

## Methods

### The cartoon development phase

Participants for the focus groups were recruited by nursing staff at a large practice in Greater Manchester that had been involved in earlier work related to the CLAHRC programme. The nurses used personal face-to-face or phone invitations, those expressing interest were contacted by the research team to obtain informed consent. This practice had a well-established register from which people with early stage CDK could be easily identified. The focus groups were held on the practice premises and led by AK and CB. We were aware that discussing CKD might raise problems due to the reluctance of primary care staff to disclose the early stages of kidney disease to patients [35], so we used the following introduction to the focus group:

‘We’ve invited you to this focus group because you are on the list (or register) your practice has of people who might be at risk of going on to develop problems with their kidneys. This register is quite a new way of doing things for the NHS and it is being done because it is thought to be important to keep a close check on you to maintain your health. We want to know what you think about that, about how you or others should be told and what sort of information might be helpful or unhelpful.’

All participants gave written informed consent prior to the focus group. The focus groups were audio-recorded and transcribed. Notes were taken during the meetings and field notes were compiled afterwards.

### Analysis

Transcripts of the focus groups were read to draw out themes related to participants’ expressed need for information and discussions around experiences of living with CKD. These themes provided a structure for the content of the guidebook. Themes on patient experience of CKD taken from existing qualitative literature helped to provide a coding framework for the analysis. Memos using Word documents were used to help create categories of information need and concerns. AK conducted the analysis and wrote the first draft of the book (revised by other members of the team). Further analysis determined where there was a collective voice emerging from the focus groups, concerning experiences or information need and this was translated into topics for potential cartoons. The team then held a series of iterative discussions to determine the final list of cartoon topics, to find quotes which best exemplified the cartoon topic and to comment on the early versions of the cartoons, some of which were redrawn several times before they were agreed on.

### The cartoon evaluation phase

Participants were recruited from those who consented to take part in a randomised controlled trial; the BRIGHT (Bringing Information and Guided Help Together) Trial [33]. The BRIGHT trial was a two-arm, patient level randomised RCT evaluating a complex self-management intervention which aimed to support the maintenance of vascular health in patients with early stage CKD. The intervention comprised the CKD guidebook and tailored access to local community resources with telephone support. Participants had a clinical diagnosis of stage 3 CKD and were identified from disease registers at GP practices. GPs then identified those they considered had been made aware of their CKD and who were able to communicate in English. They excluded patients receiving palliative care or who had reduced capacity to consent. The BRIGHT intervention significantly improved patient outcomes [paper in preparation].

**Figure 1:**
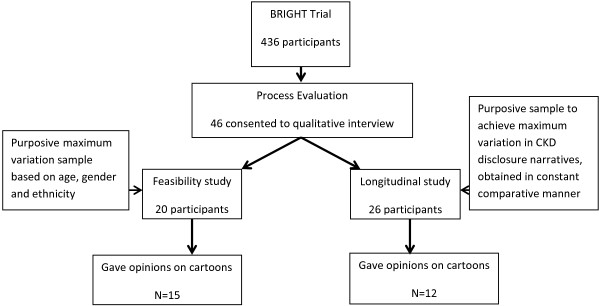
Flow chart of recruitment.

The cartoon evaluation formed part of the process evaluation of the BRIGHT trial [33]. Purposeful sampling was undertaken of those in the intervention group who gave consent to be contacted for a face-to-face interview (see Figure 1 for details). The sample aimed to include a broad range of respondents from a range of practice locations. Participants were sampled for two separate qualitative studies within the process evaluation: 1) an exploration of the feasibility of the telephone support intervention [36]; and 2) a longitudinal study to inform and explain the results of the main trial. Individuals only took part in one of these studies. All participants gave written informed consent prior to the qualitative interviews.

Questions on the cartoons formed a small part of both in-depth interview schedules and interviews with these questions took place after the participant had been given the full intervention. Participants were asked their opinion on the use of cartoons and humour in patient information and were then asked to look at the cartoons and to engage in a ‘think aloud’ discussion about each cartoon [37]. Using think-aloud is said to ‘uncover usually covert cognitive process and eliminate assumptions in analysis’ [38]. Questions used to prompt the think aloud process included: ‘what do you think about the cartoons, what are they saying?’ ‘What’s the point that’s trying to make do you think?’ ‘What does that say to you?’ ‘Just give me your interpretation of what you think it is saying.’

The interviews (conducted by GDW and RB in participants’ homes) were all audio-recorded and transcribed. The data were analysed and coded by AK to generate themes related to opinions about the cartoons and their usefulness in information.

### Analysis

The analysis was focused on the sections of the interviews where the ‘think aloud’ method was used. Opinions on each of the cartoons were compared across cases to determine how they were interpreted and understood. Charting and memos were used to highlight cartoons where there were varying interpretations and understanding. The themes which emerged were discussed with members of the team who conducted the interviews and contemporaneous field notes were referred to to ensure rigour in the interpretation.

## Results

**Figure 2:**
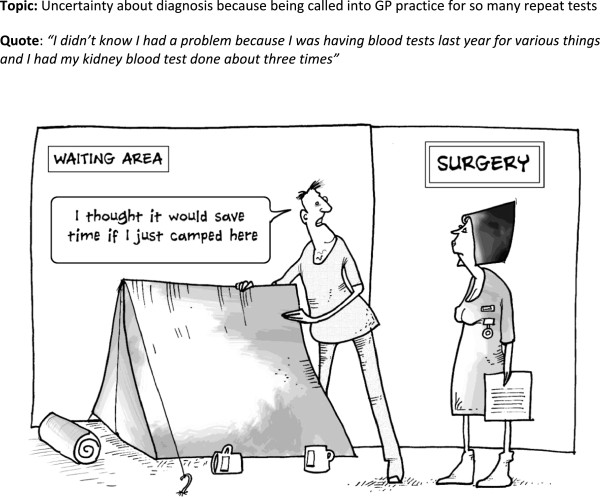
Cartoon 1.

**Figure 3:**
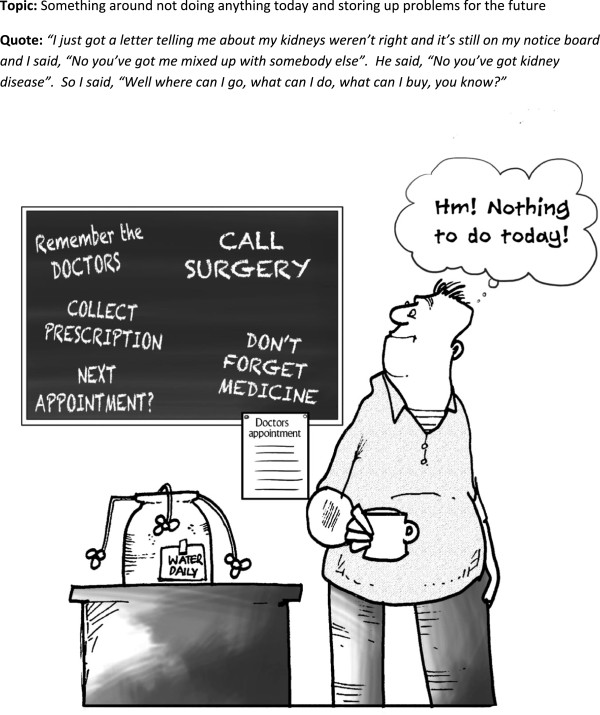
Cartoon 2.

**Figure 4:**
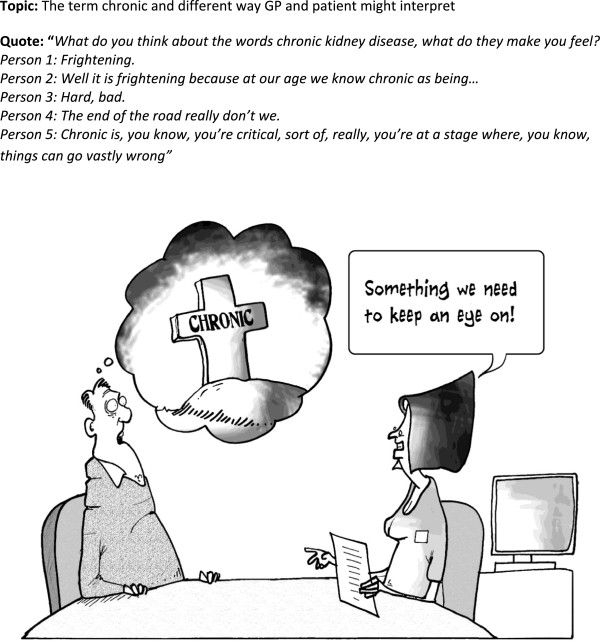
Cartoon 3.

**Figure 5:**
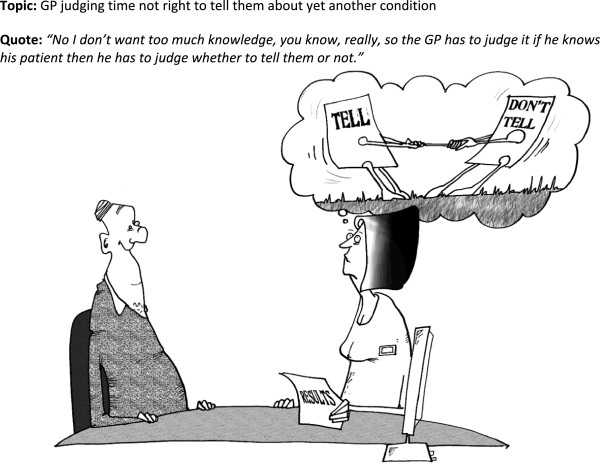
Cartoon 4.

**Figure 6:**
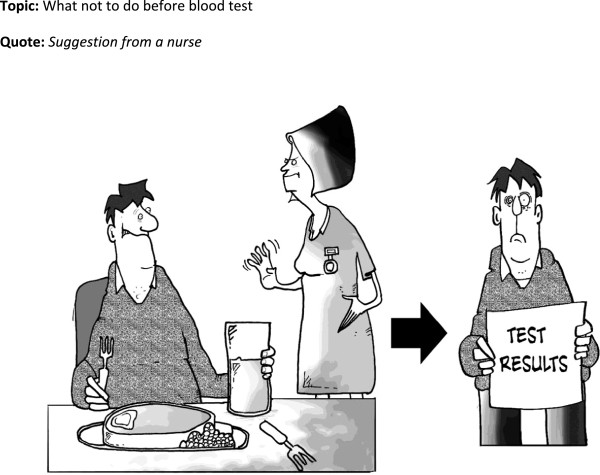
Cartoon 5.

**Figure 7:**
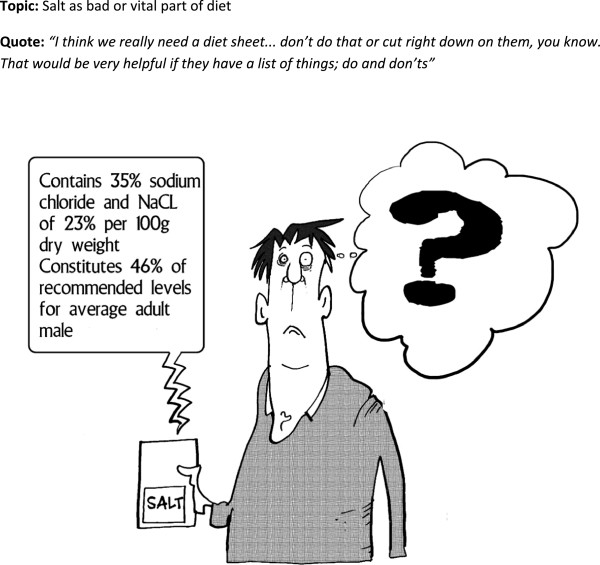
Cartoon 6.

**Figure 8:**
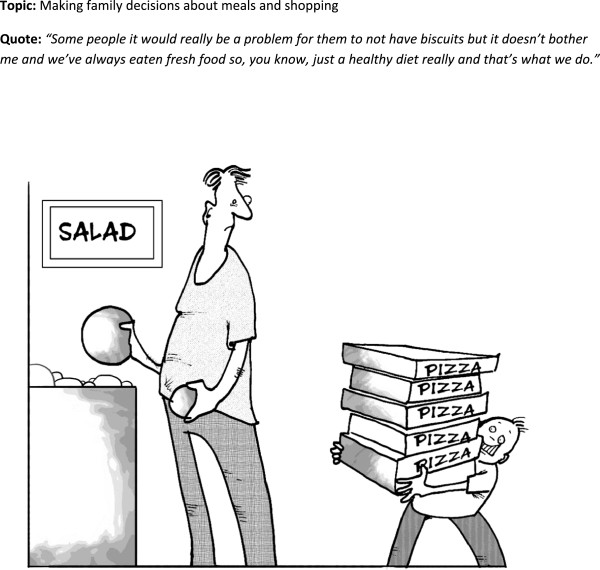
Cartoon 7.

**Figure 9:**
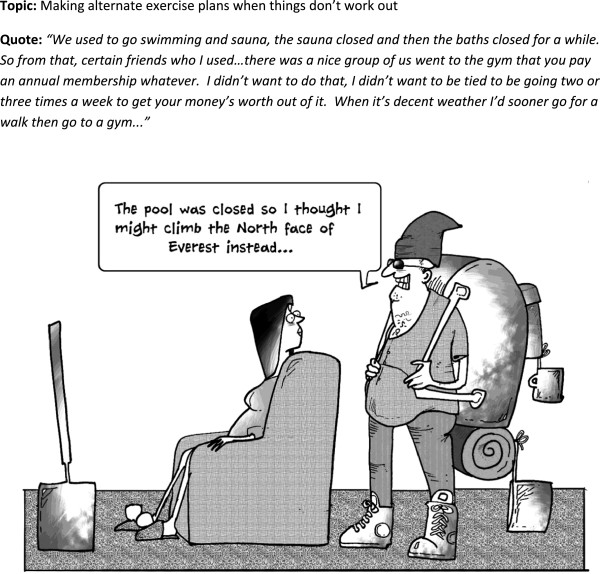
Cartoon 8.

**Figure 10:**
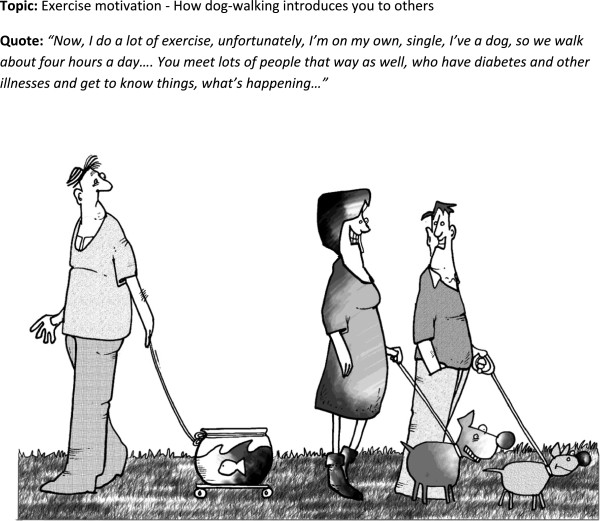
Cartoon 9.

Two focus groups were held involving 17 participants (11 were women). Figures 2- 10 outline the topics for the cartoons with the quotes that inspired the final cartoons. The topics which were included in the final guidebook concerned anxiety and uncertainty related to: getting a diagnosis; understanding the problem; feeling that facts were being withheld; and setting priorities. For example, the terminology used by health professionals generated anxiety with people thinking they were heading for kidney dialysis or early death when they heard or read the words ‘chronic kidney disease’ (Figure 4).

A total of 436 patients were recruited to the BRIGHT trial and 27 trial participants gave opinions on the CKD cartoons during the process evaluation interviews (15 from the feasibility study and 12 from the longitudinal study). Eight were male, the age range was 57 to 85 and 16 had no educational qualifications.

The evaluation of the cartoons from the ‘think aloud’ process led to themes concerning: feelings invoked by cartoons; interpretation; and adding value.

### Feelings invoked by cartoons

There were a range of views on the cartoons: outright hostility; indifference; amusement; recognition; sources of enlightenment; and as incentives to action. In general, they were viewed as lightening the tone of the information and were not thought to be upsetting.

*‘No, I think it's an illustration and, if it, if it can just lighten it a bit I think that, because there's people out there who could get really worried, whereas if you, on a cartoon it, you've got a visual picture, you know, it's, it's visual and it also might just lighten the mood.’ (id 484)*The risk of upsetting people is an important consideration and cartoons can be challenging if they provide a visual confrontation with a concern that an individual might not have wanted to consider such as debility or death. The tombstone cartoon (Figure 4) was intended to challenge people’s understanding of the word ‘chronic. Most, but not all, understood the cartoon and it prompted reflection and discussion on the meaning of ‘chronic’ and its significance (particularly in the context of the BRIGHT trial and the discussion about the CKD disclosure process). For some, the use of the term chronic was overwhelmingly emotive as the connotations were so negative, so the use of the tombstone in the cartoon had a doubly negative impact and compounded rather than resolved the miscommunication problems. A couple found the picture inappropriate. This cartoon did result in discussions with the interviewer and for some, this allowed the ‘penny to drop’ and they came to a belated understanding of the meaning.

‘The word chronic, when she came up with that we went crikey…You tend to think as the word chronic as deadly and it isn't. It's, and I've learnt in the last six weeks that it means no, it's long term. And the doctor's reassuring that by saying, 'no, it's just something we've got to keep an eye on.' And that's exactly what she said.’ (id 484)

R1 ‘that’s that chronic one isn’t it? When you mentioned the word, like I said to you chronic kidney problem… you think uh-oh when’s my time up. And that’s what that’s telling me. …But [the baseline interviewer] was the one who actually pointed out chronic doesn’t necessarily mean that you’re going to die.’

I: No. Not necessarily of that condition.

R1: Of that…exactly.

I: Yeah.

R1: Oh that’s what it’s telling you there.

I: Yeah. Well…

R1: Oh okay (id 473)

One outlier was hostile to cartoons in general and felt they prevented people taking real health problems seriously.

*‘You’re trying to get something across to somebody that can actually be quite serious. Or if you make it a cartoon and funny, they don’t even deal with it, do they, because it’s just a bit of fun, it’s not serious.’ (id 477)*There were practical problems for some people for who found the cartoons too small to allow them to see details. This led to some frustration and reading the captions and thought bubbles was a particular problem with the salt cartoon (Figure 7). Some got more out of the cartoons by reading the adjacent written text.

### I don’t get it: interpreting cartoons

Not everyone ‘got’ what the cartoons set out to depict and some of the cartoons were found difficult to interpret. Interestingly, many offered alternative explanations to that intended which made more personal sense. Cartoons present layers of meaning and can be construed as subversive, so the reading of them can trigger off different ideas for people. In using cartoons, we wanted to show the uncertainties and frustrations of life with CKD as well as to amuse, invoke recognition and promote reflection.

Interpretation was clearly dependent on context and people were being asked to talk about the cartoons in the middle of an interview about their experiences of being in a trial and receiving an intervention. The one cartoon everyone was clear about was that depicting a GP considering whether to tell the patient the CKD diagnosis (Figure 5). There was a real relevance to this cartoon because of the process of recruitment to the trial. Patients were eligible for the trial if they had an established diagnosis of early stage kidney disease and if the practice considered this had been disclosed. However, for many, the first time they became aware of their diagnosis was when their practice contacted them to invite them to take part in the trial. This resulted in anxiety or anger for some people and was a focus of an earlier part of the interview. There was though, recognition of the GP’s dilemma in disclosing information and some reflected on family members or friends for whom disclosure would be the wrong option. But for many the cartoon invoked an immediate response that they always wanted to be told the truth and had a preference for wanting to know about their condition. The latter has considerable significance in a context where it is seen as legitimate in some circumstances to withhold information about a CKD diagnosis from the patient [35].

‘R: Well, they may not be telling you exactly what you…in store for you, to a certain extent [laugh].

I: Mm. And why do you think they might not do, or do that?

R: To a certain extent, they might not want to alarm me. But there, on the other hand, I would prefer to know.’ (id 555)

Differing interpretations were offered for several cartoons, in particular the one with the tent in the GP surgery (Figure 2) and the test results one (Figure 6). For example, rather than provoking reflections on the tests, uncertainties and time taken to obtain a diagnosis, the tent cartoon was generally interpreted in relation to their NHS experiences of having to wait a long time for appointments or in the waiting room prior to consultations. It brought out rather an assertive element and a view that with the growing problems in the NHS you have to stake your claim and demand your rights to care. So the cartoon promoted a feeling of shared experience in adversity and troubled times.

‘When I first saw the tent one… you know the way the national health is going at the minute? It was like if I don’t get this up now then I’m gonna lose my place in the queue tomorrow.’ (id 473)

The test results cartoon (Figure 6) did not work well, most offered alternative interpretations around diet restrictions for CKD. This was the one cartoon that did not emerge as a result of a direct patient quote. The only people to get this were those who read the surrounding text which demonstrates how, for some, cartoons can be part of a multi-modal information strategy.

‘R: Well, he was enjoying a nice hearty meal and now he’s upset. Yeah. It’s, oh, tests can be affected by eating meat before a blood test. No, I didn’t know that.

I: Oh, you got that from the text around the cartoon…

R: Yeah.’ (id 111)

Some of the cartoons had layers of meaning. Most interpreted the salt cartoon (Figure 7) as the need to monitor and reduce salt levels in diet which was one layer of meaning. A few understood the additional message of the frustration in interpreting labels and this provoked recognition and reflection.

‘He’s read all of the things but he’d be like me…I don’t read any of the labels when I buy stuff. Because if I did, I’m just as confused. Because I don’t know what half the words mean. It’s like they’re in Latin or something to me, so I don’t bother.’ (id 566)

The blackboard cartoon (Figure 3) was related to by most people, mainly through reflecting on their personal experience of putting things off and the implicit burdens associated with help-seeking. Some people had problems interpreting this; there was probably too much going on in the small cartoon. Alternative interpretations were that it reflected people’s hectic and busy lives and the added burden of managing GP visits was too much. One managed to link this to the first cartoon with the tent (Figure 2) and added another layer of meaning by musing that this was why some people did not turn up to appointments.

*‘I’ve taken my tablet late today, after six years I still forget. Yeah, and I’ve got lots of appointments to make and I end up doing nothing [laughing],…Is it something like, you could put off till tomorrow what you could do today [laughing].’ (id 111*)

I: …can you relate to what they’re trying to say?

R: Yes, it’s very hard to get into the doctors to get an appointment.

And then they do get people that don’t turn up for appointments.

I: …so they’re linked aren’t they?

R: Yes.

I: Do you like them? Do you like what they’re trying to show?

R: Yes. Well it’s true…it’s what happens! (id 524)

### Added value or added confusion?

For some, looking at and thinking about the cartoons provided added insight into their condition and possibilities of engagement as a result. They helped to highlight the uncertainties around CKD for both patients and clinicians. The cartoons which invoked this best were the ‘tell don’t tell’ one (Figure 5) and the ‘chronic’ one (Figure 4) and they did make some reflect that they needed to be more assertive in their dealings with GPs to ensure issues were more clearly explained.

‘R: Well, that's a dilemma, isn't it? Perhaps that's why we should tell the doctor we want to know everything. We should get out there and say tell us everything, keep nothing back.

I: Do you think that, that's the way that it should work?

R: I think it should. I think that the patient decides for themselves, but if you don't know. I mean, like I've got diabetes like but nobody's said to me over the years…I've been to the doctor, I had three kids, putting weight on, gone to the doctor's about my weight. No one turned around and said, right, listen, you carry on, you'll end up with diabetes. Nobody's ever said that in all the years.’ (id 436)

Confusions arose when people tried to read the cartoons too literally, for example in the salt cartoon (Figure 7), the caption was exaggerating the complexity of food labels and was not supposed to be taken as advice, but some attempted to interpret it this way.

I: …and he’s got a big question mark coming out of his head, and then we’re told that the label on the salt packet says, ‘Contains 35 per cent sodium chloride and NACL of 23 per cent per 100 grams dry weight, constitutes 46 per cent of recommended levels for average adult male.

R: I wouldn’t know what to make of that. Yeah. I think that did puzzle me. It confuses between the whole packet quantity and what a spoonful would be. The…NACL, and , that mix might be right, but the average person wouldn’t understand why anyway. (id 483)

## Discussion

The development and dissemination of written information is insufficient to meet patient needs which are complex and multi-faceted in the area of long-term conditions. The way in which information is delivered is of importance [1]. Recently the complexity of the generation, receptivity and use of information has been highlighted by notions of health literacy which views the personal resources of patients as assets that require nurturance, development and expression [27]. Additionally research has shown how individuals utilize 'personal experiences' information in a variety of ways to support mundane as well as complex health decision-making. This does not supersede the need for 'clinical facts' which are identified by patients as relevant and necessary for inclusion in resources. The later evidence has been a feature of the development of resources discussed in this paper [39].

The inclusion of bespoke cartoons based on experiential and lay knowledge and narratives together with making use of metaphor and humour which are known agents of engagement in health matters, is one means of addressing the health literacy agenda in the arena of long-term condition management. The standardised process we developed has meant the cartoons have been well-received by patients and clinicians and suggests that they are a medium through which engagement can be initiated and harnessed. The discussion with the interviewer suggested that this may become more explicit as part of a participative dialogue. A unique aspect of this work has been the use of focus groups to ensure patient participation in the generation of the information and cartoons. The development process has been based on evidence about the relevance and salience of lay knowledge (as well as clinical knowledge) for patients [5] and the acceptability and utility of using the guidebooks in practice. However, whilst the books have been shown to be effective [40], the specific impact of the cartoons themselves is harder to measure. Williams and Cameron [10] point out that although there is evidence and theory surrounding verbal communication and the consequences of poor communication, less is known about the use of visual images and that without theory or empirical evidence, there is the potential for creating images which might cause harm.

The evaluation exercise used for the CKD guidebook highlighted the potential advantages and some of the pitfalls of using cartoons in patient information. The method of development was intended to ensure that the cartoons were based on the experiences and concerns of patients so that most would recognise the situations (albeit exaggerated). These cartoons were designed primarily to amuse, which they seemed to do, as well as to invoke recognition and reflection to provide reassurance that the uncertainties and day to day problems of living with long-term conditions in general and CKD in particular are common to all.

The lack of hard outcome measures is a limitation of using the think aloud method to evaluate the impact of cartoons. The CKD cartoons were presented in an information booklet which formed part of a package of self-management support in a randomised controlled trial. The intervention led to significantly improved patient outcomes [paper in preparation]. Although the trial was not designed to measure the specific impact of the cartoons it is in accord with evaluations of other resources using similarly developed and formatted cartoons [19]. The benefits of using think aloud are that it provides insight into the immediate impact of cartoons, indicates how they are interpreted and in conjunction with cartoons appears to be a useful method of getting people to think about their condition from a different and illuminating position. However, it is possible that embedding the cartoon related questions within broader interview agendas might have led to patients being more reflective or attuned to meaning than normal, particularly where there were emotional discussions around disclosure earlier in the interview.

In general, the use of cartoons was approved of by the target audience – but the question is do they add value to the information and what is the nature of the added value? Our evidence shows that cartoons can assist reflection and the need to take action, but they can be misinterpreted or taken too literally. They are useful as part of a wider theoretical approach to information development, participation and dissemination in self-management support.Some of the cartoons were better understood than others. The personal relevance and the context appear to be important factors in this; the ‘tell, don’t tell’ cartoon (Figure 5) was universally understood and appreciated. Cartoons were found helpful in providing insight where there was personal recognition of the dilemmas or incongruous circumstances depicted. There was evidence of ‘light bulb’ moments of understanding and these could be built on by health professionals who want to engage people more in the management of their conditions. Cartoons can be challenging as well and the difficult emotional responses some pictures evoke could be utilised to help people adjust to their condition. Misinterpretation is an interesting finding. In some cases it means the cartoon is inappropriate and this can only be determined by field-testing cartoons with the appropriate patient groups. In other cases, different interpretations to those anticipated, exposes the complexities and the often hidden feelings concerning life with a long-term condition which could be empowering for patients.The types of humour which have emerged from this process can be mapped on to the prevailing theories. People with the symptomless early stages of kidney disease were very anxious about what the diagnosis meant, with initial fears about serious consequences and this maps, to some extent, onto incongruity humour. Cartoons created for general amusement tend to draw on incongruity humour to make people laugh, but questions arise over whether it actually helps to confront people with a cartoon reflecting and depicting such incongruities (such as that used in the kidney book for Figure 4). The symbol of the tombstone did not upset the majority and did help a few gain deeper understanding, although it did compound the mental equating of ‘chronic’ with death for a few.

We have used cartoons in a patient-centred way with the topics generated directly by patients’ accounts rather than from health professionals’ traditional biomedical concerns. Cartoons could be akin to ‘weak ties’ described as helping people construct a sense of moral acceptability around illness management [41]. This fits with the purpose of using patient information to empower and engage people as outlined by Dixon-Woods [5]. Many previous attempts to convey information about self-management have failed because they tend to reflect biomedical concerns and use a mechanistic model of communication. Sufferers of long-term conditions are portrayed as passive and open to influence and manipulation in the interests of a biomedical agenda (such as adherence or compliance with medication [4,5]). The development of cartoons fits more within a second approach which is less salient and nascent and more recent in origin. This second approach has been associated with an agenda of patient focus and democratization, and its orientation towards patients. The approach we have developed and termed ‘the WISE approach’ [32], seeks to bring together evidence-based medicine and lay knowledge discourses. Drawing on a wider set of resources such as the use of cartoons contributes to this process through developing a rigorous and theoretically informed approach to patient engagement and information.

## Conclusion

The use of humour and cartoons to engage and motivate people with self-management is a consideration which is untapped by conventional theories and an approach which has the potential to improve the narrative elements and tailoring of information to support self-management [4]. Cartoons were shown to affect morale and potential future behaviour and further work is needed to build on this.

## Competing interests

The authors declare that they have no competing interests.

## Authors’ contributions

AK and AR conceived and designed all the qualitative studies; CB, RB, GD-W contributed to the design of the CKD qualitative study and collected data for the CDK cartoon evaluation. AK drafted the paper and performed the analysis. All authors read and approved the final manuscript.

## Pre-publication history

The pre-publication history for this paper can be accessed here:

http://www.biomedcentral.com/1472-6963/14/60/prepub
